# Pulmonary Nontuberculous Mycobacteria Infection in Bronchiectasis: A Narrative Review of Current Status and Future

**DOI:** 10.1002/hsr2.70749

**Published:** 2025-04-23

**Authors:** Masaki Fujita

**Affiliations:** ^1^ Department of Respiratory Medicine, Faculty of Medicine Fukuoka University Hospital, Fukuoka University Fukuoka Japan

**Keywords:** host susceptibility, macrolides, *Mycobacterium avium* complex, sex

## Abstract

**Background and Aims:**

Pulmonary nontuberculous mycobacteria (NTM) infection and bronchiectasis are two distinct respiratory conditions, but bronchiectasis and pulmonary NTM infections are closely associated. NTM can cause bronchiectasis. However, bronchiectasis can create a favorable environment for NTM colonization and exacerbate the progression of NTM. Managing both conditions typically requires a comprehensive approach that addresses infection and the underlying structural lung damage.

**Methods:**

To perform this review, the author retrieved and assessed relevant articles related to NTM and bronchiectasis that have been published to date from databases, including PubMed/MEDLINE, Scopus, and Google Scholar.

**Results:**

In this review, the close relationship between pulmonary NTM and bronchiectasis is described from the viewpoints of diagnosis, epidemiology, *Pseudomonas aeruginosa*, host susceptibility, females and NTM, and treatment.

**Conclusion:**

Timely diagnosis and management of NTM infections, especially in individuals with underlying risk factors, are essential to prevent disease progression and improve the quality of life of affected individuals.

## 1 Introduction

Pulmonary nontuberculous mycobacteria (NTM) is caused by various species of mycobacteria other than *Mycobacterium tuberculosis* and *Mycobacterium leprae*. These infections primarily affect the lungs and can lead to a range of respiratory symptoms, such as chronic cough, fatigue, and shortness of breath. Common NTM species include *Mycobacterium avium* complex (MAC), *Mycobacterium abscessus*, and *Mycobacterium kansasii*. The diagnosis of NTM infections typically involves sputum cultures and radiological imaging, such as chest X‐rays or computed tomography (CT) scans [1, 2]. Bronchiectasis is a chronic lung condition characterized by the irreversible dilation and damage of the bronchi in the lungs, resulting in the accumulation of mucus, which can lead to a persistent cough, increased susceptibility to respiratory infections, and reduced lung function. Bronchiectasis is caused by a variety of factors, such as ciliary dyskinesia, structural lung abnormalities, and autoimmune diseases. Idiopathic NTM infections can contribute to the development of bronchiectasis. When NTM organisms colonize and infect the lung tissue, they lead to inflammation and scarring of the airways. This chronic inflammation and damage to the bronchial walls can result in bronchiectasis over time. Additionally, bronchiectasis itself creates a favorable environment for the growth of NTM bacteria because the damaged airways may not effectively clear mucus and bacteria from the lungs [3, 4, 5].

## 2 Clinical Similarities Between NTM and Bronchiectasis

Diagnosing NTM infections in patients with bronchiectasis can be challenging because the symptoms of NTM overlap with those of bronchiectasis. Bronchiectasis and NTM lung disease often share similar symptoms, such as chronic cough, increased sputum production, shortness of breath, and fatigue. These overlapping symptoms can make diagnosing and managing both conditions challenging. Moreover, the presence of bronchiectasis can complicate the course of NTM infection and lead to more severe disease. An accurate diagnosis often requires a combination of clinical evaluation, imaging studies such as CT scans, and microbiological testing of respiratory samples. Specific diagnostic tests, such as sputum cultures and molecular techniques, are necessary to confirm the presence of NTM in these cases. The American Thoracic Society (ATS), European Respiratory Society (ERS), European Society of Clinical Microbiology and Infectious Diseases (ESCMID), and Infectious Diseases Society of America (IDSA) guidelines included clinical, radiographic, and microbiologic criteria for diagnosing pulmonary NTM disease. Both pulmonary or systemic symptoms and radiologic criteria, such as nodular or cavitary opacities on chest radiography or high‐resolution CT that shows bronchiectasis with multiple small nodules, are required. In addition, positive culture results from at least two separate expectorated sputum samples are needed to confirm pulmonary NTM diseases [2]. When NTM infections persist and are not properly treated, they can cause lung damage over time, potentially leading to bronchiectasis. NTM‐related bronchiectasis is often observed in individuals with certain risk factors, such as a history of NTM lung infections, chronic obstructive pulmonary disease, cystic fibrosis, and a compromised immune system [6].

**Figure 1:**
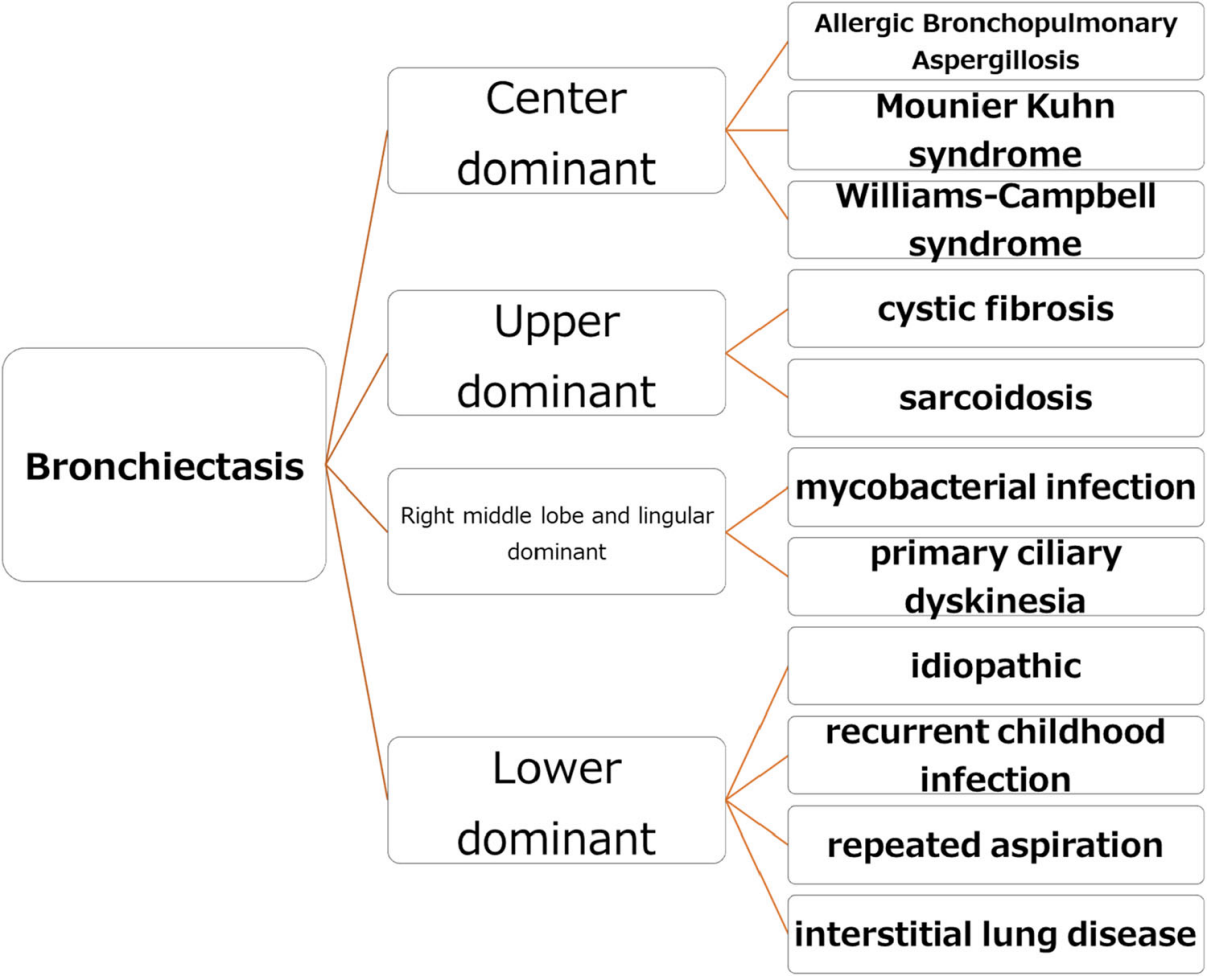
Localization of bronchiectasis and fundamental diseases.

Importantly, there is no bacteria‐specific pattern in CT findings. However, bronchodilatation of the lingular and middle lobes is frequently observed in NTM, and the upper lobe localization is characteristic of cystic fibrosis. The relationship between NTM and improper angles of the lingular and right middle lobe might be important in these diseases. Central bronchiectasis (dilation of the proximal bronchi rather than peripheral bronchi) is characteristic of allergic bronchopulmonary aspergillosis. An upper dominant pattern indicates cystic fibrosis and a lower dominant pattern indicates recurrent infection of childhood (Figure 1) [3, 4, 5]. Lee et al. [7] compared the lengths, diameters, and angles of the right middle lobe and lingular bronchi of 100 healthy subjects with normal chest CT with those in 50 individuals with NTM lung disease that involved the right middle lobe and lingular segment. The subjects were matched for sex, age, height, and body weight. They found that the right middle lobe and lingular bronchi angles were significantly greater in healthy subjects than in those with NTM lung disease. Additionally, the orifice of the right middle lobe bronchus was more narrowed than other bronchi. These findings suggest that clearing out any distal secretions from the right middle lobe and lingular bronchus is difficult [8].

Observing the clinical course of the disease is important. NTM infection in patients with bronchiectasis can lead to worsening symptoms, more frequent exacerbations, and progressive lung damage over time. At an early stage, the diagnosis of pulmonary NTM infection in patients with bronchiectasis is difficult because of the similarities in symptoms and the requirement for repeated cultures and careful evaluation. Therefore, consultation with a pulmonologist or infectious disease specialist experienced in managing these conditions is often necessary to establish a definitive diagnosis and develop an appropriate treatment plan. Treatment of bronchiectasis and NTM infection may be required to manage the dual condition effectively. The ATS and the IDSA have published guidelines for the diagnosis and management of NTM lung disease, including in patients with bronchiectasis. These guidelines outline specific criteria and recommendations for diagnosis, such as the duration and frequency of positive cultures [2].

## 3 Incidence of Pulmonary NTM in Bronchiectasis

Among the types of bronchiectasis, excluding idiopathic bronchiectasis, bronchiectasis after infection due to mycobacterial infection and pneumonia in childhood is the most common [9]. A systematic review of patients treated for tuberculosis reported that bronchodilation occurred in 35%–86% of patients, as shown by chest CT [10]. The frequency of bronchiectasis after tuberculosis varies across regions and tends to be more common in Asia than in Europe and the United States [11]. According to the Indian registry of non‐cystic fibrosis bronchiectasis (NCFB), 780 of 2195 (35.5%) patients had post‐tuberculosis bronchiectasis [12]. Nine of 147 (6.1%) patients with NCFB were reported at a single facility in Japan [13], and 31.1%, 12.0%, and 16.0% were reported in Singapore, Taiwan, and China, respectively. These findings indicated that the proportion of bronchiectasis in Japan is low [14, 15]. However, in Japan, pulmonary NTM is frequently associated with NCFB, and registry studies have shown that NTM lung disease is complicated by NCFB in 74% of patients (Asakura, personal communications). A systematic review by Zhou and colleagues showed that ~10% of NCFB was associated with NTM lung disease [16]. However, a large United States registry study reported that 63% of 1826 patients with NCFB had a history of NTM lung disease or infection [6]. Determining how many NTM cases develop bronchiectasis is difficult but should be clarified in the future.

## 4 The Relationship With *Pseudomonas aeruginosa*

In clinical practice, residual bronchiectasis as a sequelae after treatment for NTM lung disease is difficult to treat, and *Pseudomonas aeruginosa* (*P. aeruginosa*) infection also increases during and after treatment [17]. There is no apparent difference between *P. aeruginosa* infection in NTM with bronchiectasis and that without bronchiectasis. *P. aeruginosa* is a colonizing bacterium, but recently, the microbiome has been attracting attention. Disturbances in the microbiome can cause an imbalance in the immune system. Additionally, over‐activation of immunity can lead to chronic inflammation of the respiratory tract, partly due to the involvement of host genetic predisposition [18]. Therefore, bronchiectasis is considered to be an immune‐mediated inflammatory disease. Chalmers and Chotirmall stated that, regardless of the underlying disease, the pathology is persistent inflammation, and its control is the basis of treatment. The pathogenesis of bronchiectasis consists of four factors: immune inflammation of the respiratory tract, destruction and deformation of the airways and lungs, dysfunction of the epithelium and cilia, and colonization and proliferation of bacteria. These factors are intricately intertwined (vortex concept). Conventional treatment of bronchiectasis is not successful because only one of these factors is treated, and a strategy that treats two or more factors concurrently is necessary [19, 20]. Recent immunological studies have shown that macrophages react to bacteria that settle in the respiratory tract, causing neutrophil‐based inflammation, which damages the airway wall and dilates the bronchi [21, 22, 23, 24]. NTM produces fibronectin‐attached proteins, which impairs the airway epithelium. Chemical mediators, such as interleukin‐12 from macrophages, tumor necrosis factor‐α from natural killer cells and CD4‐positive T lymphocytes, interferon‐γ, and granulocyte macrophage‐colony stimulating factor are thought to form the basis of granulomatous inflammation [25].

## 5 Host Susceptibility

Human leukocyte antigen (HLA) has different host immune responses to NTM. HLA‐A26 has been reported as a poor prognostic factor in pulmonary MAC [26]. Japanese studies have also shown that HLA‐A33 and HL‐DR6 are associated with infection in pulmonary MAC [27]. Many of these cases were female, but whether HLA is associated with actual sex differences is currently unclear. However, because MAC is common in middle‐aged and older Japanese females without underlying diseases, there may be some host‐related factors related to the onset of this disease. Shojima and colleagues compared 19,651 microsatellite markers distributed throughout the human genome in blood from 300 patients with healthy controls, and reported the possibility of involvement of MHC Class I chain‐related A genetic polymorphisms with pulmonary MAC [28]. Chen et al. [29] found the TKK/hMps1 gene by whole‐exome sequencing; studies on TTK/hMps1 have exclusively focused on its role in tumorigenesis, though the contribution of TTK/hMps1 to bronchiectasis, bacterial, mycobacterial, or fungal infections, and overall progression to pulmonary disease is currently unknown. Impaired DNA damage repair in host cells induced by defects in the TTK gene may provide a beneficial environment for bacterial proliferation or may compromise local innate immune responses, allowing infections to cause disease. Bronchiectasis‐susceptibility genes might be similar to NTM‐susceptibility genes [29]. Recently, several genome‐wide association studies have been reported. Cho and colleagues reported that the 7p13 genetic variant might be associated with susceptibility to NTM pulmonary disease in the Korean population by altering the expression level of STK17A, a proapoptotic gene [30]. Namkoong and colleagues reported single‐nucleotide polymorphisms in calcineurin‐like EF‐hand protein 2 (*CHP2*) [31]. However, additional evidence is required to establish the function of these specific genes in NTM susceptibility. Szymanski and colleagues examined genetic variants in patients with NTM, their unaffected family members, and a control group. They proposed that NTM infection is a multigenic disease in which combinations of variants across gene categories with environmental exposures increase susceptibility [32]. These reports indicated that bronchiectasis lesions may be more likely to precede NTM lesions. However, Asakura and colleagues recently reported that NCFB exhibits distinct proximal and distal bronchiolar muco‐obstructive disease [33]; additionally, NTM RNA was found in the cavitary region of bronchiectasis, suggesting the possibility that NTM lesions could arise first in some patients with bronchiectasis.

## 6 Females and NTM

After menopause in middle‐aged and older females, NTM can develop in the middle lobe and lingular area; this is known as Lady Windermere syndrome [34]. The clearance of the middle lobe and lingular is poor anatomically. Therefore, colonization and subsequent NTM infection can easily occur. Females have worse clearance than males. In addition, menopause attenuates the function of sex hormones and alveolar macrophages, which play a protective role premenopause. Therefore, NTM and other bacteria that form colonies become potentially active. This physical stimulus may lead to bronchiectasis [35]. Ciliary motility of the fallopian tube (fallopian duct) decreases in postmenopausal females, suggesting that female hormones are one of the factors that regulate ciliary movement in the body [36]. Recently, progesterone was reported to reduce the frequency of ciliary beat frequency of airway epithelial ciliary movement [37]. This finding suggests that female hormones are detrimental to the respiratory tract epithelium. However, in this report, progesterone concentrations appeared to be much higher than normal, and whether female hormones impair ciliary movement in the respiratory tract is unclear [37].

## 7 Treatment

Treatment of bronchiectasis can be divided into the chronic stable phase and the exacerbation phase. In addition to the treatment of the primary disease, sputum regulators and rehabilitation are important in the chronic stable phase, and long‐term macrolide administration is recommended for cases of frequent exacerbations [9, 38]. In European guidelines [9], the use of inhaled antibacterial drugs is recommended for bronchiectasis cases in which *P. aeruginosa* settles and exacerbates repeatedly. However, in Japan, inhaled antimicrobials other than tobramycin for cystic fibrosis are not covered by insurance. In the event of exacerbation of bronchiectasis, 14‐day antibiotic administration is recommended, but the administration period may need to be shortened or extended in each case. Inhaled corticosteroids and bronchodilators may be used during chronic stability, but evidence is scarce, and routine use is not recommended at this time. These treatment options alone are insufficient; there are cases in which frequent exacerbations are repeated, and intravenous treatment must be used in a small portion of patients with bronchiectasis. Macrolides have been shown to have a variety of effects, such as suppressing the migration and activation of inflammatory cells, the production of reactive oxygen species, biofilm production by *P. aeruginosa*, and airway secretion [39]. A large randomized, controlled study of azithromycin and erythromycin showed a reduction in the frequency of acute exacerbation of bronchiectasis and an improvement in the quality of life [40, 41, 42, 43]. However, macrolides did not improve lung function [44]. Based on these findings, the use of macrolides is spreading in Europe and the United States. However, macrolide resistance is also spreading [45]. Azithromycin and clarithromycin are key drugs for treating pulmonary NTM; these drugs should not be used as single agents for possible pulmonary NTM in patients with bronchiectasis [2].

Managing NTM lung disease in the presence of bronchiectasis requires a multidisciplinary approach involving pulmonologists, infectious disease specialists, and respiratory therapists. The treatment of NTM typically involves a combination of antibiotics to target NTM species (azithromycin, ethambutol, rifampicin, and aminoglycoside) and therapies aimed at controlling bronchiectasis symptoms, improving airway clearance, and preventing further lung damage [2]. The guidelines for NTM treatment have been published by the ATS, ERS, ESCMID, and IDSA. A new treatment for refractory MAC disease has also been introduced. Liposomal amikacin has been reported to be effective [2]. Liposomal amikacin is also effective for *P. aeruginosa* and could be a new remedy for NTM with bronchiectasis, aiming to manage bronchiectasis symptoms and prevent further lung damage. Treatment can be prolonged and may require multiple antibiotics, but successful treatment can improve symptoms and slow the progression of bronchiectasis. Airway clearance techniques can help reduce mucus buildup in the damaged airways, and bronchodilators may be prescribed to alleviate airflow obstruction and improve breathing. In some cases, immunosuppressive medications may be used to manage inflammation. Improvement in bronchiectasis‐related symptoms and stabilization of bronchiectatic changes on imaging are observed when patients with NTM‐related bronchiectasis receive appropriate treatment. These findings provide evidence of a causal relationship between the two conditions; while there is a clear association between pulmonary NTM infections and bronchiectasis, not all patients with NTM infections will develop bronchiectasis, and its severity and progression can vary widely among individuals. Early diagnosis, appropriate treatment of NTM infections, and close monitoring are essential for managing bronchiectasis in patients with NTM and preventing further lung damage. Additionally, individual cases may vary, and treatment decisions should be made in consultation with healthcare professionals according to the patient's specific condition and clinical evaluation.

## 8 Conclusion

NTM infection can lead to bronchiectasis, which is a chronic lung condition characterized by damaged airways. Conversely, bronchiectasis may be complicated by NTM. Timely diagnosis and management of NTM infections, especially in individuals with underlying risk factors, are essential to prevent the progression of this disease and improve the quality of life for affected individuals.

## Author Contributions

**Masaki Fujita:** project administration, writing – review and editing.

## Conflicts of Interest

The author declares no conflicts of interest.

## Transparency Statement

The lead author Masaki Fujita affirms that this manuscript is an honest, accurate, and transparent account of the study being reported; that no important aspects of the study have been omitted; and that any discrepancies from the study as planned (and, if relevant, registered) have been explained.

## Data Availability

The author has nothing to report.
